# Blood Protozoan Infection Disrupts Ovarian Follicular Development in Dairy Cattle: A Mechanistic Link to Reproductive Failure

**DOI:** 10.1002/vms3.71115

**Published:** 2026-07-21

**Authors:** Most. Mousumi Akter, Md. Sadequl Islam, Md. Ashraful Islam, Md. Mahmudul Hasan, Rakibul Islam, Md. Mominul Islam, Md. Gausur Rahman, Md. Shafiqul Islam, Anup Kumar Talukder, Md. Bazlar Rashid

**Affiliations:** ^1^ Department of Physiology and Pharmacology Hajee Mohammad Danesh Science and Technology University Dinajpur Bangladesh; ^2^ Department of Anatomy and Histology Hajee Mohammad Danesh Science and Technology University Dinajpur Bangladesh; ^3^ Department of Pathology Sher‐e‐Bangla Agricultural University Dhaka Bangladesh; ^4^ Department of Pathology and Parasitology Hajee Mohammad Danesh Science and Technology University Dinajpur Bangladesh; ^5^ Department of Pharmacology Faculty of Veterinary Science Bangladesh Agricultural University Mymensingh Bangladesh; ^6^ Department of Gynecology Obstetrics and Reproductive Health Faculty of Veterinary Medicine and Animal Science Gazipur Agricultural University Gazipur Bangladesh

**Keywords:** anaemia, dairy cattle infertility, haemoglobin, haemoprotozoa, histopathology, iron metabolism, ovarian follicle

## Abstract

**Background:**

Haemoprotozoan infections are a pervasive threat to livestock health in tropical regions, yet their specific impacts on reproductive function remain unidentified.

**Objective:**

This study tested the hypothesis that blood protozoan infection induces anaemia and disrupts ovarian follicular dynamics, thereby contributing to infertility.

**Method:**

Forty cows (*n* = 40) with reproductive complaints were examined, and blood samples were analysed for protozoan infection and haemoglobin (Hb) concentration. Ten cyclic cows without any reproductive problems were served as controls. Ex vivo assessments were performed on reproductive tracts (*n* = 30) collected from an abattoir, followed by histopathological examination of ovarian tissues. A complementary rabbit (*n* = 10) model was conducted to assess haematological and iron metabolic changes across the oestrous cycle.

**Result:**

Among 30 symptomatic cows, 100% tested positive for blood protozoan infection, with distribution rates of 53.3% (8/15) in anoestrus, 40.0% (4/10) in repeat breeding and 20.0% (1/5) in conception failure. Infected cows exhibited significantly lower Hb levels (6.6 g%) compared to non‐infected controls (10.6 g%). Cows with Hb ≤6.5 g% exhibited rudimentary ovaries, small to medium follicles and follicular cysts, whereas those with Hb ≥7 g% showed normal ovarian morphology. Histopathology confirmed cystic degeneration, fibrosis and aberrant follicular structures in infected cows. The rabbit model showed a significant decline in Hb and disruptions in serum iron levels during the anoestrus phase, reinforcing the link between iron metabolism and reproductive cyclicity.

**Conclusion:**

Our findings demonstrate that subclinical blood protozoan infection severely impairs ovarian health through anaemia‐induced hypoxia and metabolic disturbances, establishing a direct link to infertility.

## Graphical abstract

Subclinical haemoprotozoan infection disrupts RBC integrity, and that leads to decreased Hb level and excess free iron in plasma. That subsequently impairs ovarian follicular development and thereby builds up a mechanistic link to reproductive failure in dairy cattle.

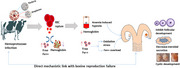

## 1 Introduction

Reproductive efficiency is the cornerstone of profitability in dairy farming, influencing milk yield, replacement costs and herd sustainability (Walsh et al. 2011). In developing nations like Bangladesh, infertility accounts for significant culling losses, yet the aetiological complexity—encompassing genetics, nutrition and disease—often obscures specific causative factors (Plaizier et al. 1998; Shahiduzzaman et al. 1999). Among infectious agents, haemoprotozoan parasites (e.g., *Theileria annulata*, *Babesia bigemina*, *Anaplasma marginale*) are endemic, causing substantial morbidity and economic loss through anaemia, weight loss and reduced productivity (Kocan et al. 2004; Velusamy et al. 2014). Globally, the prevalence of bovine haemoprotozoan infections ranges from 15% to 85% in tropical and subtropical regions, with the highest burdens reported in South Asia, Sub‐Saharan Africa and Latin America (Aktas et al. 2019; Sivakumar et al. 2014).

The pathophysiological consequences of haemoprotozoosis extend beyond overt clinical illness. These infections induce a profound haemolytic anaemia, depleting haemoglobin (Hb) and altering critical serum minerals such as iron, copper, zinc and calcium (Das et al. 2022; Ganguly et al. 2015). These elements are indispensable for reproductive physiology: Iron is central to oxygen transport and cellular respiration; zinc and copper are cofactors for steroidogenesis and antioxidant enzymes; calcium mediates oocyte activation and uterine contractility (Khillare et al. 2007; Gałęska et al. 2022). Anaemia compromises oxygen delivery to metabolically active tissues, including the ovary, where follicular development and oocyte maturation are highly sensitive to hypoxia (Lim et al. 2021). Recent evidence indicates that hypoxia‐inducible factor 1‐alpha (HIF‐1α) accumulates in granulosa cells under low oxygen tension, altering expression of steroidogenic enzymes and promoting apoptosis (Jansen et al. 2021). Furthermore, iron deficiency alone has been shown to disrupt oestrous cyclicity and reduce conception rates in experimental models (Li et al. 2014).

Despite the known systemic impact of haemoprotozoan infections, a direct, mechanistic link between these infections, induced anaemia and specific ovarian pathological changes in cattle remains poorly documented. Although several studies have reported haematological alterations in infected cattle (Shaukat et al. 2019; Modi et al. 2015), none have systematically examined ovarian histopathology in relation to Hb status. Most studies focus on haematological alterations or general reproductive ‘failure’ without elucidating the ovarian morphological and histological sequelae. Furthermore, conflicting evidence exists: some researchers report no significant association between subclinical haemoprotozoan infection and fertility parameters (Mekonnen et al. 2019), suggesting that host nutritional status, parasite load and co‐infections may modulate reproductive outcomes. This gap hinders the development of targeted interventions.

We hypothesized that blood protozoan infection induces significant anaemia, which, in turn, disrupts follicular development and ovarian integrity, leading to the clinical manifestations of infertility. Therefore, this study was designed with the following objectives: (i) to determine the prevalence of blood protozoan infection in dairy cows presenting with reproductive disorders; (ii) to correlate infection status with Hb levels and clinical reproductive symptoms; (iii) to assess the impact of infection and associated anaemia on ovarian gross morphology and histoarchitecture; and (iv) to validate the relationship between Hb/iron status and reproductive phase using a synchronized rabbit model.

## 2 Materials and Methods

### 2.1 Study Design, Period and Location

**FIGURE 1:**
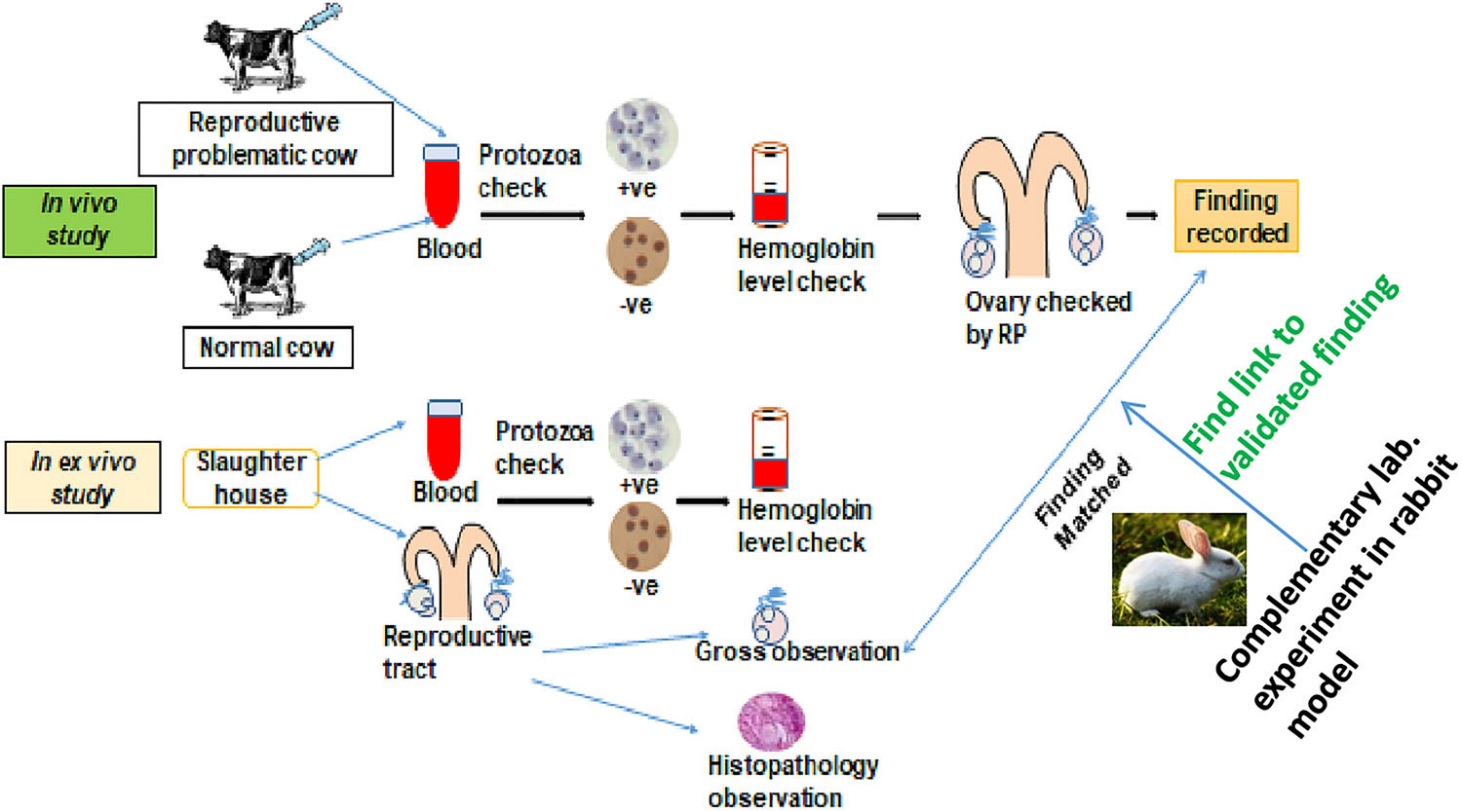
Schematic overview of experimental design. This study involved two phases: (i) an in vivo assessment of dairy cows with reproductive issues from local farms, and (ii) an ex vivo examination of reproductive tracts collected from a local abattoir with matched findings between both approaches. A complementary experiment using rabbit model was conducted to validate the data obtained.

A cross‐sectional field study combined with ex vivo laboratory analysis was conducted from January 2023 to June 2025 at Dinajpur Sadar, Bangladesh. The study involved two phases: (i) an in vivo assessment of dairy cows with reproductive issues from local farms, and (ii) an ex vivo examination of reproductive tracts collected from a local abattoir. A complementary experiment using rabbit model was conducted to validate the data obtained (Figure 1).

### 2.2 Animal Selection and Sample Collection (Bovine)

#### 2.2.1 In Vivo Study

Forty (*n* = 40) lactating dairy cows (crossbred Holstein‐Friesian × local) with a history of reproductive failure were purposively selected from 12 commercial farms in Dinajpur Sadar Upazila (25°37′ N, 88°38′ E), a region with known endemicity for tick‐borne diseases (Rahman et al. 2018). The inclusion criteria were as follows: (i) Anoestrus is defined as the absence of oestrus signs for >90 days post‐partum based on farm records and two consecutive trans‐rectal palpations 10 days apart; (ii) repeat breeding is defined as failure to conceive after ≥3 consecutive inseminations with fertile bull semen confirmed by the artificial insemination technician; and (iii) confirmed conception failure is defined as the absence of pregnancy confirmed by trans‐rectal ultrasonography (5–7.5 MHz linear probe, Sonosite, USA) at 35–45 days post‐insemination following two successive insemination attempts. Ten normal cyclic cows (regular oestrus cycles every 18–22 days, confirmed by farm records and two consecutive palpations) were served as clinical controls. All cows underwent comprehensive clinical examination, including body condition scoring (BCS; 1–5 scale; Ferguson et al. 1994), assessment for mastitis using California Mastitis Test (CMT), and evaluation of lameness and other visible pathologies. Cows with BCS < 2.5, positive CMT or clinical signs of other systemic diseases (e.g., foot rot, chronic diarrhoea and respiratory distress) were excluded to minimize confounding factors. Blood samples (5 mL) were collected from the jugular vein into EDTA tubes (BD Vacutainer, Becton Dickinson, Franklin Lakes, NJ, USA) for analysis.

#### 2.2.2 Ex Vivo Study

Thirty (*n* = 30) ovarian pairs with corresponding blood samples (collected from the right ventricular chamber via cardiac puncture immediately following exsanguination) were collected post‐slaughter from Bahadur Bazar, Dinajpur abattoir, which processes culled dairy cows from approximately 15 surrounding commercial farms. Cows sent to this abattoir were primarily culled for low milk production, advanced age or reproductive failure, thus representing a population with potentially higher baseline reproductive pathology. Ovaries were immediately placed in cold (4°C) phosphate‐buffered saline (PBS, pH 7.4) and transported to the laboratory within 2 h. For histological examination, ovaries were fixed in 10% neutral buffered formalin within 4 h of collection to prevent autolytic changes.

### 2.3 Measurements of Hb Concentrations

Hb concentrations were determined using Sahli's acid–haematin method (Sahli's haemoglobinometer; JSGW, India). Briefly, 20 µL of blood was mixed with N/10 HCl, allowed to convert to acid haematin for 10 min and diluted with distilled water until the colour matched a standard comparator. The concentration was read at the lower meniscus and expressed in grams per decilitre (g%). All measurements were performed in duplicate, and the average value was recorded. The coefficient of variation for duplicate measurements was <3%.

### 2.4 Diagnosis of Blood Protozoan Infection

Thin and thick blood smears were prepared, air‐dried, fixed in absolute methanol and stained with 10% Giemsa stain for 30 min. Smears were examined under a light microscope (1000× magnification, oil immersion) for the presence of intra‐erythrocytic or free *Theileria*, *Babesia* or *Anaplasma* organisms, following standard parasitological identification keys (Soulsby 1982). Each smear was examined independently by two experienced parasitologists who were blinded to the animals’ clinical status. Discordant results (observed in 4/70 smears, 5.7%) were resolved by a third examiner. Samples were considered positive if at least two examiners identified the organism.

### 2.5 Assessment of Ovarian Morphology

#### 2.5.1 In Vivo Study

Trans‐rectal palpation was performed by a single experienced veterinarian (with >10 years of dairy cattle reproductive examination experience) who was blinded to the animals' infection status and Hb levels. To ensure blinding, blood samples were coded, and Hb results were not shared with the examiner until after all ovarian assessments were completed. The examiner assessed ovarian size (normal vs. rudimentary), consistency and follicular structures (presence, size and number of follicles). Follicle diameter was estimated by comparison with known reference structures (e.g., 5 mm = pea‐sized, 10 mm = marble‐sized) as described by Pierson and Ginther (1984).

#### 2.5.2 Ex Vivo Study

Ovaries were cleaned of surrounding connective tissue, and the diameter of surface follicles was measured directly using a digital vernier calliper (±0.01 mm accuracy; Mitutoyo Corporation, Japan). Follicles were categorized as small (3–5 mm), medium (6–9 mm) or large (10–20 mm) (Kor et al. 2013). Ovarian size was classified as rudimentary (length <15 mm, width <10 mm, underdeveloped) or normal on the basis of gross visual inspection using standard bovine ovarian morphometry (Ireland et al. 1984).

### 2.6 Histopathological Examination

Ovarian tissues from abattoir samples (with known Hb and infection status) were fixed in 10% neutral buffered formalin for 48 h. Tissues were processed through graded ethanol dehydration, cleared in xylene and embedded in paraffin wax. Sections of 5 µm thickness were cut using a rotary microtome, mounted on slides and stained with haematoxylin and eosin (H&E) using a standard protocol (Luna 1998). Slides were examined under a light microscope by a veterinary pathologist blinded to sample identity for pathological changes (e.g., cystic follicles, fibrosis, haemorrhagic corpora and oocyte degeneration). A semi‐quantitative scoring system (0 = absent, 1 = mild, 2 = moderate, 3 = severe) was used to grade histopathological lesions.

### 2.7 Complementary Rabbit Experiment

To isolate the effect of haematological changes on reproductive cyclicity, 10 (*n* = 10) mature, healthy female New Zealand White rabbits (weight 2.5–3.0 kg, age 6–8 months) were synchronized using intramuscular injection of 20 IU eCG (Equine Chorionic Gonadotropin; MSD Animal Health, the Netherlands). Blood samples were collected at three phases: pre‐synchronization (baseline), oestrus (48–56 h post‐eCG; confirmed by vulvar colour and receptivity to a teaser male) and induced anoestrus (post‐ovulation; 72–96 h post‐eCG). Hb and serum iron levels were measured using Sahli's method and a colourimetric ferrozine‐based assay using commercially available kits (Randox Laboratories, UK), respectively.

### 2.8 Statistical Analysis

Data were analysed using SPSS software (Version 22.0). Descriptive statistics are presented as mean ± standard error of the mean (SEM). Differences in Hb levels between infected and non‐infected groups, and among clinical symptom groups, were analysed using one‐way ANOVA followed by Tukey's post hoc test. The chi‐square (*χ* ^2^) test was used to assess associations between categorical variables (infection status and clinical signs). For the rabbit experiment, paired *t*‐tests were used to compare parameters between oestrus and anoestrus phases. *p* value of <0.05 was considered statistically significant.

## 3 Results

### 3.1 Association Between Blood Protozoan Infection and Clinical Reproductive Symptoms

**FIGURE 2:**
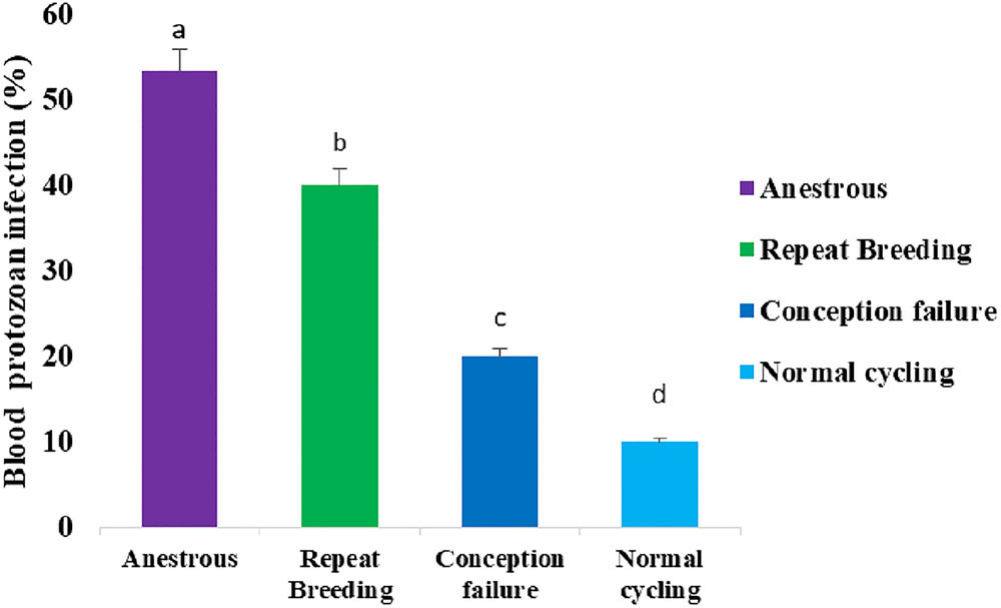
Prevalence of blood protozoan infection in dairy cows with different clinical reproductive symptoms. Blood smears from 40 cows (15 anoestrus, 10 repeat breeding, 5 conception failure and 10 normal cyclic) were examined via Giemsa staining. Bars represent the percentage of animals within each clinical group that tested positive for blood protozoa (*Theileria*/*Babesia*/*Anaplasma* spp.). Different lowercase letters (a, b, c) above bars indicate statistically significant differences between groups (*p* < 0.05, one‐way ANOVA with Tukey's post hoc test). NC, normal cyclic.

Microscopic examination confirmed blood protozoan infection in 30 of 30 (100%) cows presenting with reproductive complaints from the in vivo study. Note: The total in vivo cohort comprised 40 cows (30 symptomatic + 10 normal cyclic controls). The distribution of infection among specific symptoms was highly significant (*p* < 0.05, *χ* ^2^ test): Among the 30 symptomatic cows, 53.33% (8/15) of anoestrus cows, 40% (4/10) of repeat breeders and 20% (1/5) of conception failure cases were infected. Notably, only 10% (1/10) of normal cyclic cows were infected (Figure 2). Infection status was determined by microscopic examination of Giemsa‐stained blood smears (described in Section 2.5), with positive identification on the basis of visualization of intra‐erythrocytic *Theileria*, *Babesia* or *Anaplasma* organisms at 1000× magnification. Mixed infections (*Theileria* + *Anaplasma*) were observed in 6/30 (20%) of positive samples.

### 3.2 Hb Levels in Relation to Clinical Symptoms and Infection Status

**FIGURE 3:**
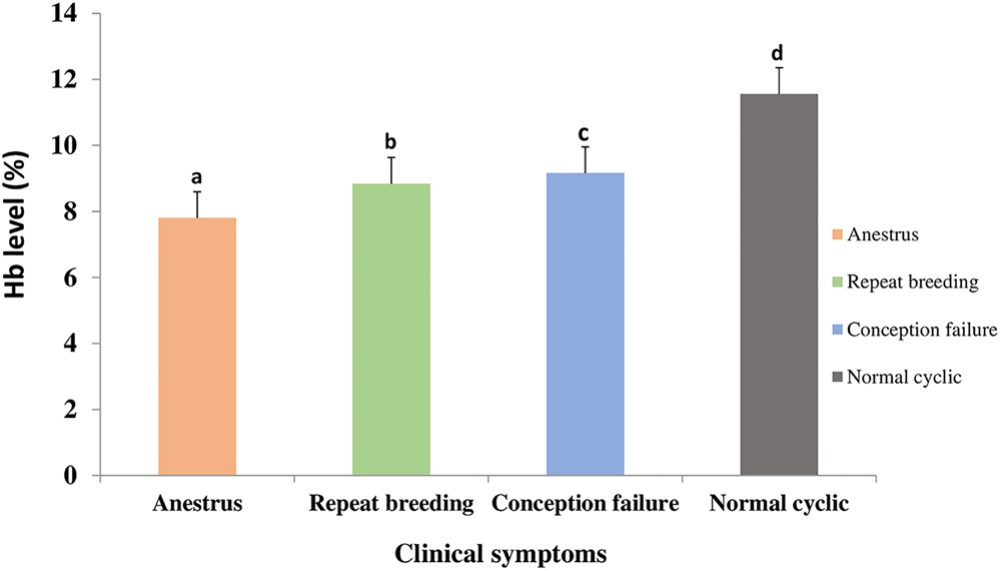
Mean haemoglobin (Hb) concentration in dairy cows presenting with different reproductive problems. Haemoglobin levels (g%) were measured using Sahli's method. Data are presented as mean ± SEM. Different lowercase letters (a, b, c) above bars indicate statistically significant differences between groups (*p* < 0.05, one‐way ANOVA with Tukey's post hoc test). NC, normal cyclic.

**FIGURE 4:**
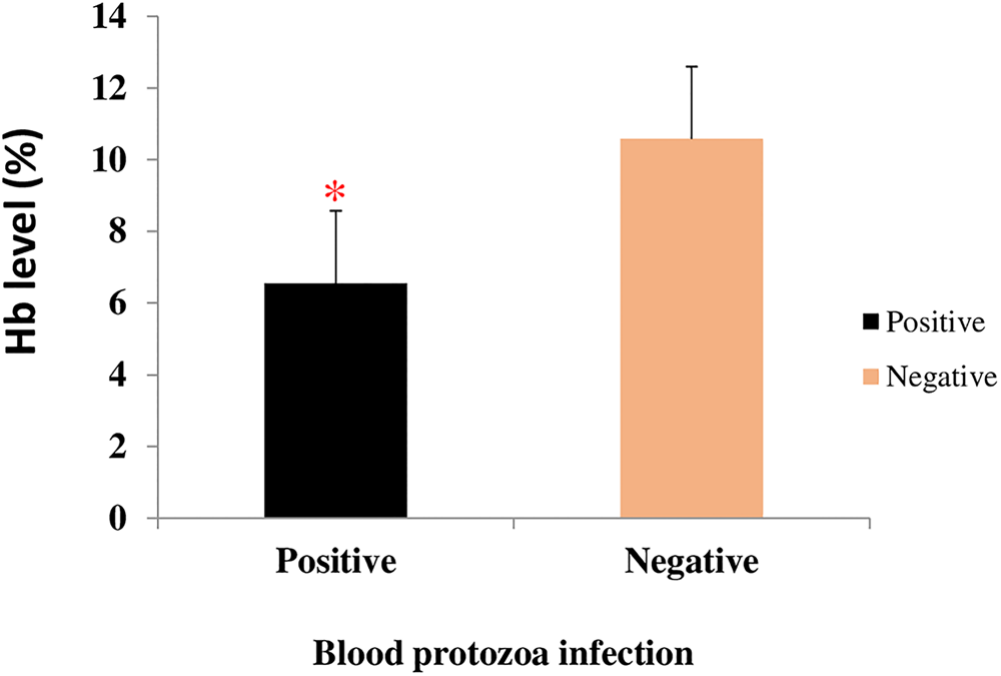
Comparison of haemoglobin levels between blood protozoa‐infected and non‐infected dairy cows. The mean haemoglobin concentration was significantly lower in the infected group (*n* = 14) compared to the non‐infected group (*n* = 26). Data are mean ± SEM. *p < 0.05 (Student's *t*‐test).

Hb levels varied significantly across clinical groups (*p* < 0.05). The mean Hb was lowest in anoestrus cows (7.8 ± 0.4 g%), intermediate in repeat breeding (8.84 ± 0.5 g%) and conception failure (9.16 ± 0.6 g%) groups, and highest in normal cyclic cows (11.56 ± 0.3 g%) (Figure 3). Critically, protozoa‐infected animals had a mean Hb of 6.55 ± 0.3 g%, significantly lower (*p* < 0.01) than the 10.58 ± 0.4 g% observed in non‐infected animals (Figure 4). The species of protozoan infection influenced Hb levels, with *Theileria*‐positive animals showing the most severe anaemia (5.98 ± 0.4 g%), followed by *Anaplasma* (6.45 ± 0.5 g%) and *Babesia* (7.12 ± 0.6 g%), though these differences were not statistically significant.

### 3.3 Impact of Hb Level on Ovarian Health (In Vivo)

Rectal palpation revealed a clear dichotomy in ovarian health based on Hb levels (Table 1). Cows with low Hb (5.5–6.5 g%) predominantly had rudimentary ovaries (42.5%), small (32.5%) or medium (22.5%) follicles, and a 20% incidence of cystic ovaries. No cows in this Hb range had large follicles or normal ovarian size. In contrast, cows with higher Hb (7–10 g%) exhibited normal ovarian size (57.5%), large follicles (45%) and an absence of cystic structures. Intermediate Hb levels (6.6–6.9 g%) showed mixed phenotypes, suggesting a possible threshold effect at approximately 7.0 g%.

**TABLE 1:** Effect of haemoglobin level on ovarian health parameters assessed in vivo (*n* = 40).

Hb (g%)	*n*	Follicle size (%)	Cystic ovary (%)	Ovary size (%)
5.5–6.5	17	Small (32.5), medium (22.5)	Present (20)	Rudimentary (42.5)
6.6–6.9	8	Mixed (small/medium/large)	Present (5)	Mixed (normal/rudimentary)
7–10	15	Large (45)	Absent (0)	Normal (57.5)

### 3.4 Gross and Histopathological Observations of Ovaries (Ex Vivo)

**FIGURE 5:**
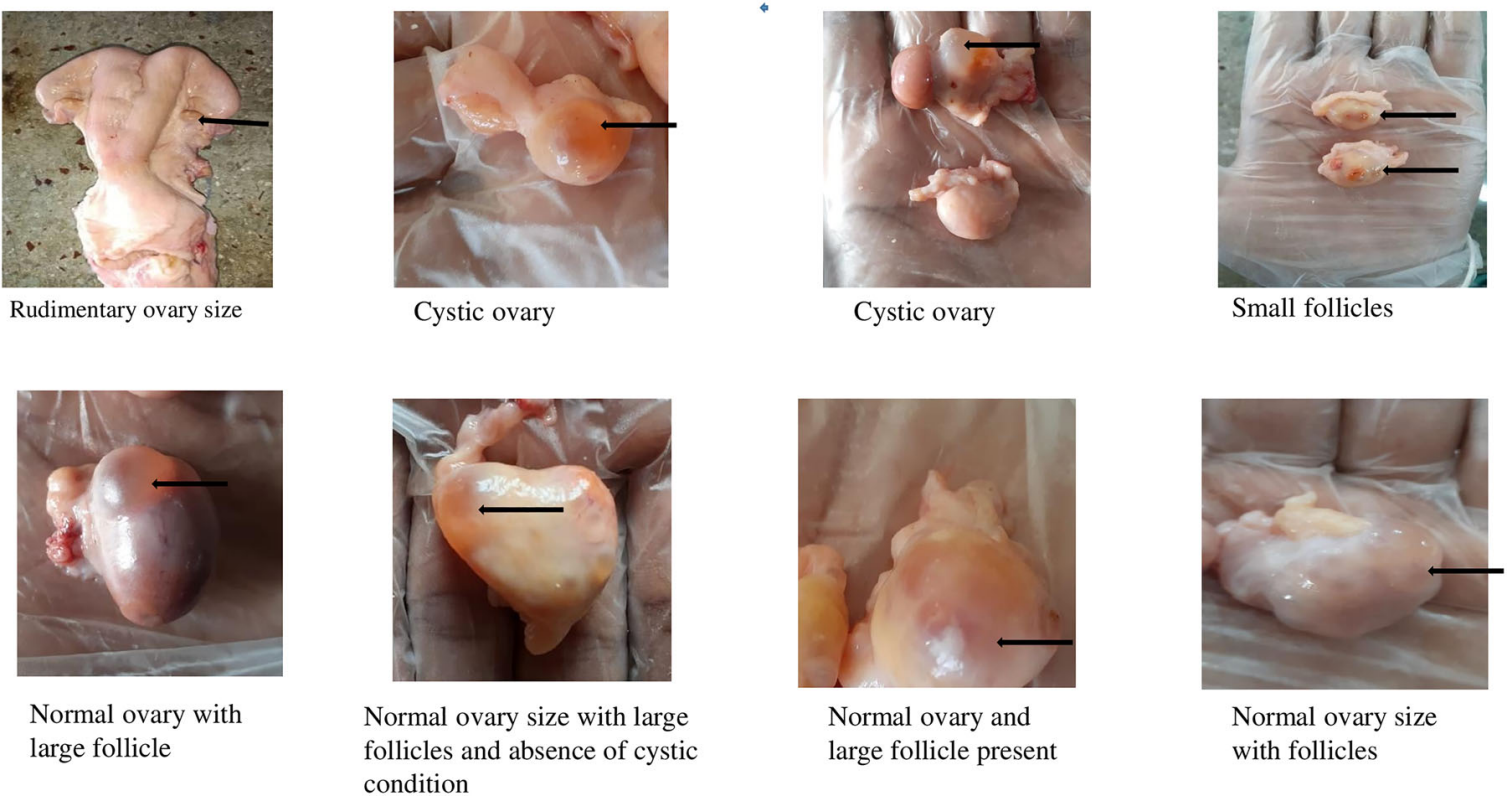
Gross observation of ovary (ex vivo). According to gross data, there were 33.3% small, 26.67% medium and 40% large follicular sizes. Ovarian size was 66% normal, 33% rudimentary and 23.33% cystic. When haemoglobin levels are between 5.5 and 6.5 g%, ovary health shows rudimentary size, small or medium follicular size, and cystic ovary; in contrast, when haemoglobin levels are between 7 and 9 g%, ovary health was normal, with large follicular size and no cystic condition.

Gross examination of abattoir‐derived ovaries (*n* = 30) corroborated the in vivo findings. Ovaries from animals with Hb ≤ 6.5 g% were often rudimentary (33%) and cystic (23.3%), with a high proportion of small (33.3%) and medium (26.7%) follicles. Those with Hb ≥ 7 g% were normal (66%) and contained large follicles (40%) (Figure 5). The correlation between Hb level and follicle size was statistically significant (Spearman's rho = 0.72, *p* < 0.001).

**FIGURE 6:**
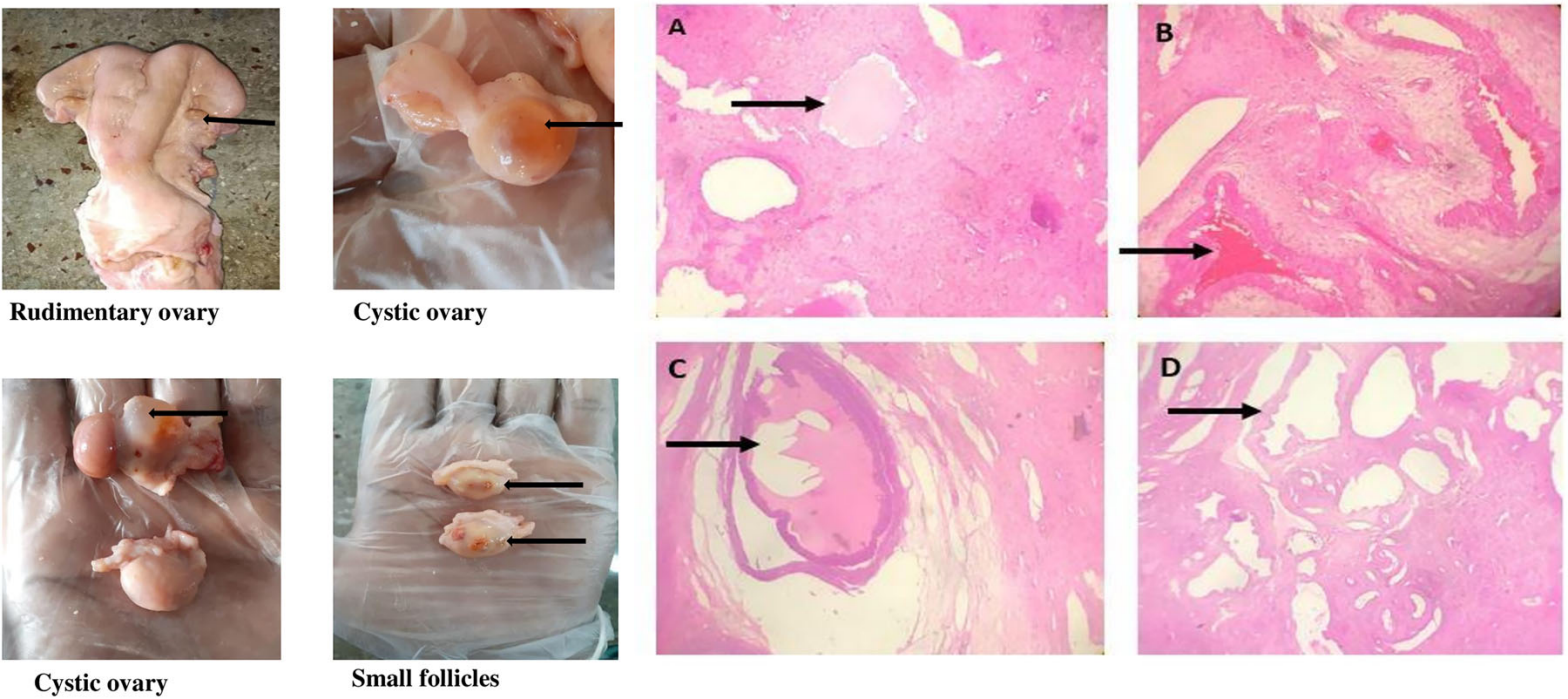
Histopathological examination (H&E stain, 400× magnification): (A) follicular cyst formation with accumulation of eosinophilic fluid and surrounded by one or two layers of compressed granulosa cells. (B) Corpus haemorrhagicum surrounded by fibrous tissues. (C) A large fluid‐filled cystic follicle surrounded by fibrous connective tissues. (D) Follicles with varying degrees of size and shape without oocyte in their centre indicating failed oogenesis.

Histopathological examination of ovaries from infected, anaemic cows revealed severe pathological alterations (Figure 6). Cows infected with the protozoan parasite showed a marked tendency to develop follicular cysts within the ovary. Specifically, 8/12 (66.7%) of ovaries from infected, anaemic cows (Hb ≤ 6.5 g%) exhibited one or more cystic follicles (>10 mm diameter with attenuated granulosa cell layer), compared to 1/18 (5.6%) of ovaries from non‐infected, normocytic cows (*p* < 0.001, Fisher's exact test). In addition, extensive structural abnormalities were observed, including ovarian fibrosis (moderate to severe fibrosis in 9/12 infected anaemic cows) and follicles of varying size that lacked identifiable oocytes (10/12 infected anaemic cows had ≥3 such structures per section) (Figure 6). In contrast, ovaries from non‐infected control animals showed normal follicular architecture with intact oocytes and healthy granulosa cell layers. These findings indicate significant disruption of normal ovarian architecture and suggest impaired folliculogenesis associated with protozoan infection.

### 3.5 Rabbit Experiment: Haematological Shifts Across the Oestrous Cycle

The rabbit model provided direct evidence of cyclical haematological changes linked to reproductive status (Table 2). A significant decline in Hb (from 12.77 ± 0.38 to 11.95 ± 0.05 g/dL; *p* = 0.003) was observed during the anoestrus phase compared to oestrus. Concurrently, serum iron levels paradoxically increased during anoestrus (133.40 ± 1.20 vs. 87.67 ± 1.37 µg/dL in oestrus; *p* < 0.001), suggesting a disruption in iron utilization for erythropoiesis possibly due to hepcidin‐mediated iron sequestration (as seen in anaemia of chronic disease). Total WBC and platelet counts were also significantly lower during anoestrus, suggesting a generalized bone marrow suppression or peripheral consumption during the anoestrus phase.

**TABLE 2:** Haematological and iron metabolic parameters in synchronized rabbits during oestrus and anoestrus phases (*n* = 10; mean ± SEM).

Parameter	Oestrus phase	Anoestrus phase	*p* value
Haemoglobin (g/dL)	12.77 ± 0.38	11.95 ± 0.05	0.003
Serum iron (µg/dL)	87.67 ± 1.37	133.40 ± 1.20	<0.001
Total WBC (cells/µL)	8803.33 ± 101.38	6000 ± 40	<0.001
Platelet count (×10^3^/µL)	216.67 ± 14.53	117.50 ± 2.50	<0.001
HCT/PCV (%)	29.00 ± 2.04	36.60 ± 0.30	0.02

Abbreviation: SEM, standard error of the mean.

## 4 Discussion

This study establishes a compelling etiopathological link between blood protozoan infection, secondary anaemia and distinct ovarian dysfunction in dairy cattle. The 100% (30/30) infection rate among infertile cows, contrasted with 10% (1/10) in cyclic controls, underscores haemoprotozoosis as a major, often overlooked, contributor to reproductive failure in endemic regions like Bangladesh. This aligns with global reports attributing significant reproductive losses to tick‐borne diseases (Rajput et al. 2005; Krishna Murthy et al. 2016). However, our infection rate is higher than the 45%–75% prevalence typically reported for subclinical haemoprotozoan infections in South Asian dairy cattle (Aktas et al. 2019; Sivakumar et al. 2014), likely reflecting our selective enrolment of animals with reproductive complaints rather than general herd sampling.

We acknowledge that the 100% infection rate in symptomatic cows may overestimate the true population attributable risk, as our control group had only 10% infection. A larger case–control study with prevalence‐matched controls would provide more precise estimates of the odds ratio for infertility associated with haemoprotozoan infection.

The central mechanistic finding is the severe anaemia (mean Hb 6.55 g%) induced by protozoan infection. Haemolysis and immune‐mediated destruction of RBCs directly deplete Hb, the critical oxygen‐carrying molecule (Shaukat et al. 2019; Modi et al. 2015). Our results demonstrate that this anaemia is not merely a bystander effect but is directly correlated with the severity of ovarian compromise. Cows with Hb levels below 6.5 g% exhibited rudimentary ovaries, arrested follicular growth (small/medium follicles), and a high prevalence of cysts. This supports the hypothesis that ovarian hypoxia, resulting from reduced oxygen delivery, disrupts the finely tuned process of folliculogenesis. The ovary, particularly the avascular follicular antrum and the metabolically active oocyte‐granulosa cell complex, is highly susceptible to hypoxic stress (Lim et al. 2021). Hypoxia can alter steroidogenic gene expression, induce granulosa cell apoptosis and impair oocyte competence, leading to follicular atresia or pathological cyst formation (Brown et al. 2015). In support of this mechanism, Jansen et al. (2021) demonstrated that culturing bovine granulosa cells under 1% O_2_ (severe hypoxia) reduced oestradiol production by 60% and increased apoptosis marker Caspase‐3 by 3‐fold compared to normoxic controls—a finding that parallels our in vivo observations of cystic follicles with attenuated granulosa layers.

The histopathological evidence provides a tangible morphological correlate to the functional impairment. The observed follicular cysts with attenuated linings, fibrous encapsulation of structures and oocyte‐deficient follicles are classic indicators of chronic ovarian insult and failed ovulation (Mijares et al. 2010). Fibrosis suggests ongoing inflammation and tissue repair, possibly driven by parasitic antigens or secondary infections. The absence of oocytes in otherwise visible follicles is a stark sign of germ cell degeneration, likely a terminal consequence of sustained metabolic deprivation. Our semi‐quantitative scoring revealed that moderate‐to‐severe ovarian fibrosis was present exclusively in infected cows with Hb < 6.5 g% (9/12, 75%), supporting a dose–response relationship between anaemia severity and tissue pathology.

Beyond hypoxia, the infection‐induced depletion of essential trace minerals (Das et al. 2022) creates a multifactorial assault on reproduction. Deficiencies in iron (required for cytochrome P450 enzymes in steroidogenesis), zinc (essential for nucleic acid synthesis and gonadotropin function) and copper (a cofactor for antioxidant enzymes like SOD) synergistically impair ovarian resilience and endocrine signalling (Khillare et al. 2007; Gałęska et al. 2022). A recent meta‐analysis by Sirotkin (2021) confirmed that deficiencies in iron, zinc and copper each independently reduce ovarian steroidogenesis by 25%–40% in mammalian models, and combined deficiencies have additive effects.

The rabbit experiment elegantly decouples infection from the haematological‐reproductive axis. The significant drop in Hb during anoestrus, accompanied by dysregulated iron metabolism (high serum iron but low Hb), mirrors the anaemia of chronic disease. This pattern—elevated serum iron with low Hb—is characteristic of inflammation‐induced hepcidin upregulation, which sequesters iron in macrophages and reduces its availability for erythropoiesis (Ganz 2019). This suggests that even in the absence of active haemolysis, disruptions in iron homeostasis can suppress erythropoiesis and potentially impair reproductive cyclicity, as seen in chronic inflammatory states (Li et al. 2014). The parallel decline in WBC and platelets during anoestrus may reflect broader systemic or energy‐conserving adaptations or could indicate bone marrow suppression secondary to chronic inflammation.

Recognizing the multifactorial nature of infertility (Walsh et al. 2011), our findings do not suggest that haemoprotozoan infection is the sole cause of reproductive failure in endemic regions. Rather, we propose that it acts as a significant, modifiable risk factor that interacts with nutritional, genetic and management‐related variables. For instance, anaemic, infected cows may be more susceptible to the negative fertility effects of heat stress or suboptimal body condition. Future research should explore these interactions using multivariate modelling approaches with larger sample sizes.

Acknowledging the relatively small sample size (*n* = 40 cows, *n* = 30 abattoir samples), our findings should be interpreted as preliminary but compelling evidence for an Hb‐ovarian health axis. The consistency of findings across two independent sample sets (field cases and abattoir‐derived tissues) and a complementary rabbit model strengthens the biological plausibility despite the modest numbers. Nevertheless, we concur with the reviewer that larger, multi‐centre prospective studies with power calculations to determine optimal sample sizes are urgently needed to validate these associations and establish Hb thresholds for clinical decision‐making. Furthermore, although our sample size was sufficient to detect large effect sizes (Cohen's *d* > 0.8; achieved power >0.80 for primary outcomes), we acknowledge limited power for subgroup analyses.

A limitation of the abattoir‐based component is the inability to obtain complete clinical histories, including parity, stage of lactation and previous disease episodes. Cows sent to slaughter may represent a selected population with chronic health issues that could independently affect ovarian morphology. However, the consistency between abattoir findings and our field data—where clinical histories were available—mitigates this concern. Additionally, we cannot exclude the possibility that concurrent subclinical diseases (e.g., mastitis, lameness or Johne's disease) contributed to the observed ovarian changes. Future studies should incorporate ante‐mortem health assessments and post‐mortem tissue collection from the same animals to better control for confounding comorbidities.

Our findings have immediate practical implications. Routine screening for haemoprotozoan infection and Hb status should be integral to infertility workups in endemic areas. Management strategies must extend beyond antiprotozoal treatment to include nutritional support for replenishing iron stores and trace minerals (e.g., iron dextran and mineral boluses). On the basis of our findings, we suggest a provisional Hb threshold of 7.0 g as a target for intervention, though prospective studies are needed to validate this cut‐off. Addressing anaemia and mineral imbalances could significantly improve ovarian recovery and subsequent fertility.

Future studies should also investigate whether treatment of haemoprotozoan infection (e.g., buparvaquone for *Theileria* and imidocarb for *Babesia*) combined with iron supplementation restores normal follicular development and conception rates. Longitudinal studies tracking infected cows from diagnosis through treatment and fertility outcomes would strengthen the causal link. Furthermore, molecular speciation of the protozoa and quantification of ovarian hypoxia markers (e.g., HIF‐1α and VEGF) would provide deeper mechanistic insight.

## 5 Conclusion

This study demonstrates that blood protozoan infection is a major, pervasive cause of reproductive dysfunction in dairy cattle, primarily mediated through the induction of severe anaemia. The resulting hypoxic and metabolic stress disrupts follicular development, promotes cystic ovarian degeneration and leads to the clinical syndromes of anoestrus, repeat breeding and conception failure. The corroborative rabbit model confirms the intrinsic relationship between Hb/iron dynamics and reproductive cyclicity. These findings advocate for an integrated herd health approach in endemic regions, combining proactive parasite control, regular haematological monitoring and targeted nutritional therapy to mitigate this hidden threat to dairy productivity.

## Author Contributions

**Most. Mousumi Akter**: writing – original draft, analysis, manuscript preparation, software. **Md. Sadequl Islam**: writing – original draft, methodology, investigation. **Md. Ashraful Islam**: writing – original draft, methodology, investigation. **Md. Mahmudul Hasan**: formal analysis, data curation. **Rakibul Islam**: formal analysis, data curation. **Md. Mominul Islam**: editing, analysis, supervision. **Md. Gausur Rahman**: editing draft, data curation. **Md. Shafiqul Islam**: reviewing, editing draft, supervision. **Anup Kumar Talukder**: reviewing and editing draft, data curation. **Md. Bazlar Rashid**: conceptualization, supervision, funding acquisition, writing – review and editing, project administration.

## Funding

The authors have nothing to report.

## Ethics Statement

All procedures involving animals were approved by the Animal Experiments Ethics Committee of Hajee Mohammad Danesh Science and Technology University (Approval No. HSTU/VAS/PPH/Ethics‐17) and complied with the Bangladesh Veterinary Council guidelines for the care and use of research animals. The rabbit experiment was conducted under the same ethical approval and followed ARRIVE guidelines (Animal Research: Reporting of In Vivo Experiments; Percie du Sert et al. 2020).

## Conflicts of Interest

The authors declare no conflicts of interest.

## Data Availability

The datasets generated and analysed during this study are available from the corresponding author upon reasonable request.
